# Necrotizing Sialometaplasia of the Hard Palate in a Patient Treated with Topical Nonsteroidal Anti-Inflammatory Drug

**DOI:** 10.1155/2016/9545861

**Published:** 2016-10-19

**Authors:** Alessandro Gatti, Emanuele Broccardo, Giuseppe Poglio, Arnaldo Benech

**Affiliations:** Unit of Oral and Maxillofacial Surgery, Maggiore della Carità University Hospital, Novara, Italy

## Abstract

Necrotizing sialometaplasia is a rare, benign, self-limiting, necrotizing process involving the minor salivary glands, mainly the mucoserous glands of the hard palate. It is thought to be the result of an ischemic event of the vasculature supplying the salivary gland lobules. Some predisposing factors such as smoking, use of alcohol, denture wearing, recent surgery, traumatic injuries, respiratory infections, systemic diseases bulimia, and anorexia have been described. Herein we present a case of necrotizing sialometaplasia of the hard palate in a patient without known predisposing factors, in our opinion, resulting from the use of topical anti-inflammatory drug. After diagnosis, the patient underwent treatment with chlorhexidine gluconate and a full palatal acrylic guard to protect the exposed bone from food residues during meals. After the sixth week the lesion regressed.

## 1. Introduction

Necrotizing sialometaplasia (NS) is a rare, benign, self-limiting, necrotizing process involving the minor salivary glands, in most cases the mucoserous glands of the hard palate. This lesion was first described in 1973 by Abrams et al. [1]. However, it may be confused with malignant lesions both histopathologically and clinically, in particular squamous cell carcinoma and mucoepidermoid carcinoma [2].

Etiologically NS is thought to be the result of an ischemic event of the vasculature supplying the salivary gland lobules. However, some predisposing factors related to these lesions have been described, including smoking, use of alcohol, denture wearing, recent surgery, other traumatic injuries, respiratory infections, systemic diseases [3–5], bulimia, and anorexia [6]. In the literature no cases of NS are described in relation to the use of anti-inflammatory drugs.

We describe a case of necrotizing sialometaplasia of the hard palate in a patient treated with topical anti-inflammatory drug, in the absence of other predisposing factors.

## 2. Clinical Report

**Figure 1:**
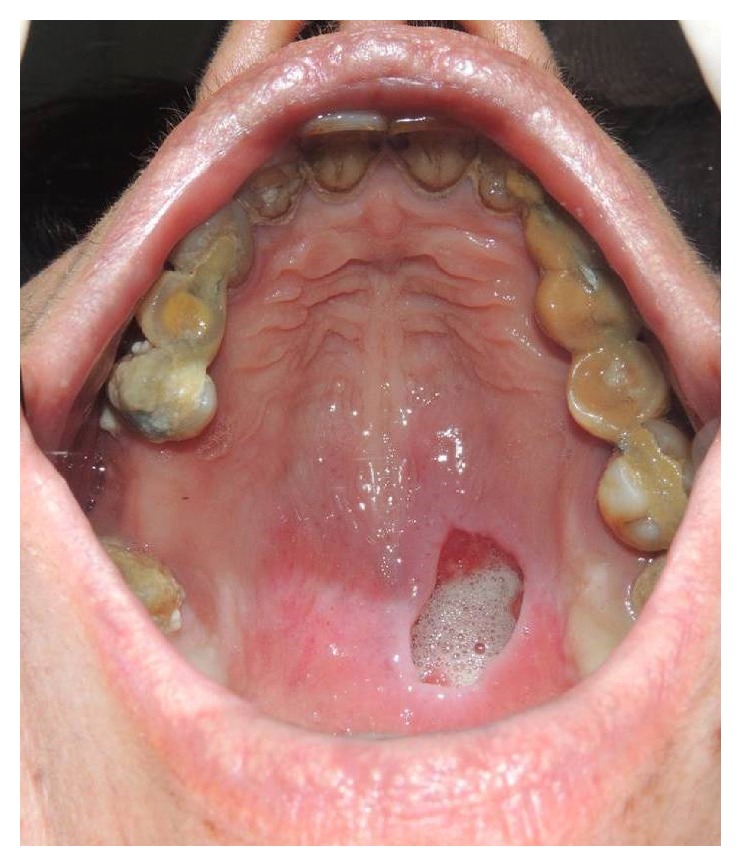
Deep crateriform ulcer of the palatal mucosa, located at the left side of the midline of the hard palate.

**Figure 2:**
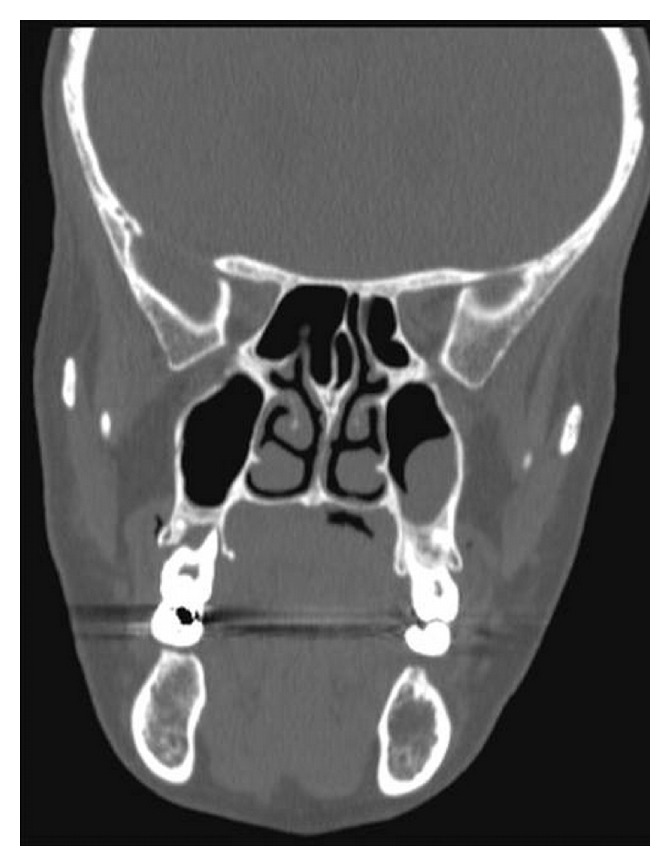
Computed tomography scan showing a mucosal lesion without bone involvement (coronal view).

**Figure 3:**
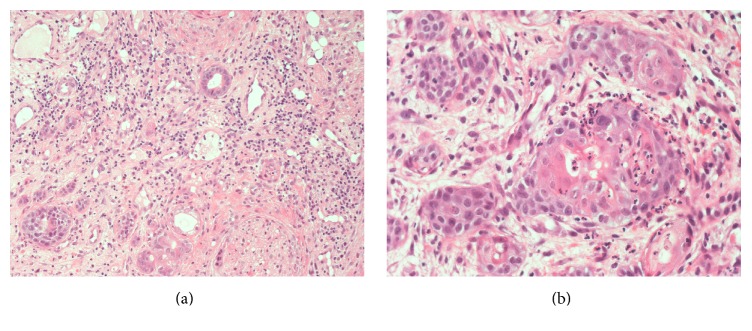
Photomicrographs of the histological specimen, showing a reactive inflammatory process involving the minor salivary glands, associated with focal necrosis of the lobules and areas of squamous metaplasia of the salivary ducts. (a) H&E, 20x magnification and (b) H&E, 40x magnification.

A 47-year-old woman presented with a painless swelling in the hard palate, which appeared 2 days before. The patient reported pain during swallowing. There was no history of alcohol intake, smoking, systemic disease, trauma, or recent surgery. The only relevant fact that we found in the patient's history was the excessive use of flurbiprofen oral spray for 5 weeks, which she reported as prevention for sore throat. No diagnosis of pharyngitis was made. The patient reported over 6 daily administration for 5 weeks. Physical examination revealed a deep crateriform ulcer of the palatal mucosa, measuring 20 mm in diameter, located at the left side of the midline of the hard palate. The ulcer appeared with sharp margins without evidence of mucosal erythema (Figure 1), and the underlying bone was exposed without any sign of infection and erosion. A computed tomography scan showed a mucosal lesion without bone involvement (Figure 2) and laterocervical reactive lymphadenopathy. An incisional biopsy of the lesion was performed under local anesthesia. The histopathological examination showed a reactive inflammatory process involving the minor salivary glands, associated with focal necrosis of the lobules and areas of squamous metaplasia of the salivary ducts (Figure 3).

**Figure 4:**
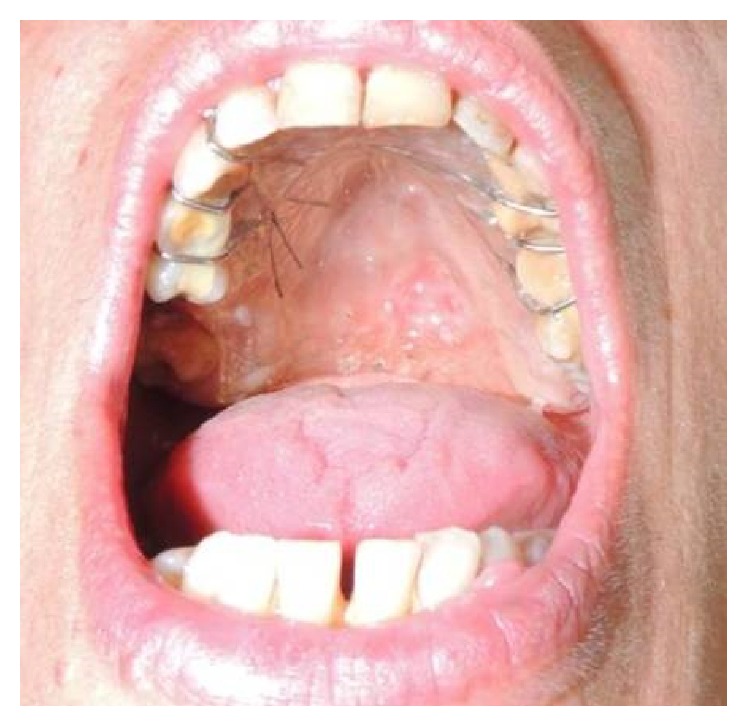
Full palatal acrylic guard lined with silicone placed to protect the exposed bone and to reduce the pain on swallowing.

**Figure 5:**
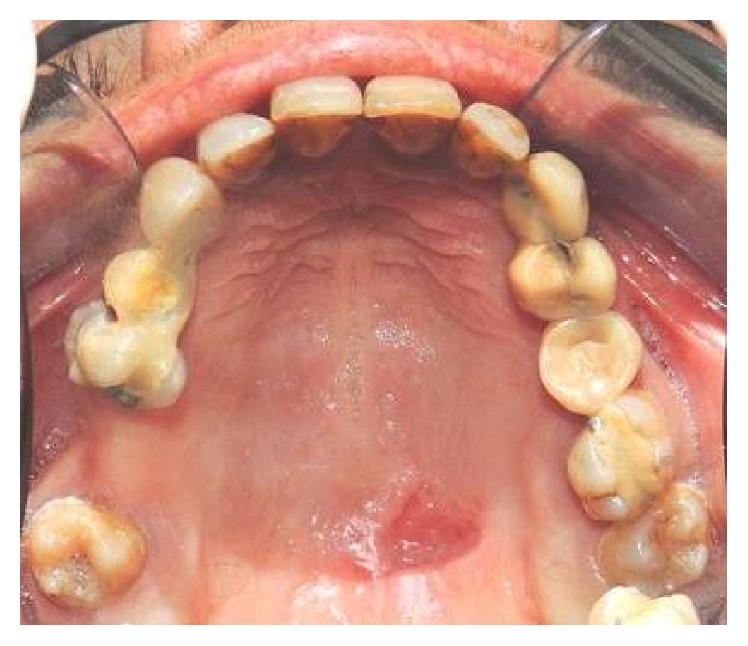
Regression of the lesion at sixth week of follow-up.

The patient underwent treatment with chlorhexidine gluconate gel 3 times a day; a full palatal acrylic guard was fabricated, lined with silicone and placed to protect the exposed bone from food residues during meals and to reduce the pain on swallowing (Figure 4). A weekly follow-up was carried out for 8 weeks. After the first week the pain during swallowing was resolved. After the sixth week the lesion regressed (Figure 5).

## 3. Discussion

Necrotizing sialometaplasia was first described in 1973, by Abrams et al. [1]. This lesion is a benign inflammatory condition which can be found at any site in the body that contains elements of salivary gland. In the literature cases of NS located at the lung and at the paranasal sinuses are reported, but most of the cases have still been reported in the oral cavity [7]. The etiology of NS has not been fully understood, but an underlying cause appears to be the gland tissue ischemia. The appearance of these lesions is believed to be related to a physicochemical or biological injury on the blood vessels that would produce an ischemic event and consequently necrosis, inflammation, and metaplasia of the ducts [4].

Anneroth and Hansen, in their study [8], have described the pathogenesis of NS, dividing it into five histologic stages: infarction, sequestration, ulceration, the reparative stage, and the healed stage.

Numerous risk factors have been described to explain the onset of local ischemia of the palate. Local trauma is considered the most frequent of them. Before extraction of maxillary teeth, the injection of local anesthetic with adrenaline to the hard palate may contribute to ischemia through pharmacologic vasoconstriction [9]. Surgery may also represent a form of local trauma, either as an accidental traumatic event during intubation or extubation or due to prolonged local pressure. Poor-fitting prostheses are also considered a predisposing factor; in fact the damage may result from a prolonged compression on the palate [7]. Alcohol, tobacco, or cocaine abuse may decrease blood flow to the mucosa, especially if these factors coexist [4]. Other possible predisposing factors cited in the literature are chemical irritation resulting from bulimia and chronic vomiting; there may be a combination of mechanical and chemical factors such as the low pH of the gastric contents and the use of fingers to mechanically induce vomiting [6]. Radiation, respiratory infections or allergies, previous adenoidectomy or surgery for other lesions, and adjacent tumours may produce compression and consequently ischemia [3].

Some systemic diseases are considered as a predisposing factor such as diabetes, HIV [10], due to reduced immunity defense, and drepanocytic anaemia, with an increase in blood viscosity that favours ischemia.

Recently Senapati et al. demonstrated that necrotizing sialometaplasia could be a manifestation of a localized vasculitis [11].

In the literature, to our knowledge, a case of NS associated with the use of topical nonsteroidal anti-inflammatory drugs has never been described.

Nonsteroidal anti-inflammatory drugs normally elicit therapeutic anti-inflammatory, analgesic, and antipyretic effects by inhibition of prostaglandins synthesis. They, for the most part, competitively inhibit cyclooxygenases, the enzymes that catalyze the synthesis of cyclic endoperoxides from arachidonic acid to form prostaglandins [12, 13].

Prostaglandins and their derivatives, such as prostacyclin and thromboxane, are involved in physiological functions such as protection of the stomach mucosa, aggregation of platelets, and regulation of kidney function. In addition, they have a well-documented pathophysiological role in inflammation, fever, and pain [13].

Prostaglandins are in fact potent mediators of inflammation that results in edema, pain, and vasodilation. The inhibition of these compounds is associated with analgesia and a reduction in inflammation [12].

Nevertheless, in 2006, Cannon et al. [14] demonstrated that prolonged use of systemic nonsteroidal anti-inflammatory drugs increases the risk of cardiovascular events. Suppression of prostacyclin and prostaglandin E2, mediated by this class of drugs, is the most thoroughly developed explanation for the cardiovascular hazard; in fact, prostacyclin is a prostanoid that acts as a restraint on mediators of platelet activation, hypertension, and atherogenesis, including thromboxane A2.

In our case the patient had abused topical flurbiprofen, a nonsteroidal anti-inflammatory drug, which also contained alcohol as excipient. The patient reported more than 6 daily doses for more than one month.

We suppose that the synergistic action of nonsteroidal anti-inflammatory drug and alcohol, acting as local microenvironmental factors on the palatal mucosa for a long time, had resulted in an ischemic event of the vasculature supplying a salivary gland.

The treatment of necrotizing sialometaplasia does not require surgery; in fact this kind of lesion is a self-limiting condition. When risk factors are removed, healing is obtained within 4–8 weeks [3]. In this case we have treated the patients with chlorhexidine gel 3 times daily to control the inflammation and, thanks to the use of a full palatal acrylic guard, the storage of food residues inside the ulcer with consequent risk of inflammation and pain has been avoided. The healing has been regularly obtained after 6 weeks.

To the best of our knowledge, this is the first case of NS associated with the use of topical nonsteroidal anti-inflammatory drug. We suppose that the prolonged use of topical anti-inflammatory drugs may be considered as a predisposing factor to NS. More extensive histopathological and clinical studies are necessary to confirm this hypothesis and to clarify the correct pathogenesis of this rare, benign disease.
